# Where Should Orphaned and Separated Children and Adolescents Live: Comparing Institutionalized- and Family-Based Venues in Kenya

**DOI:** 10.1001/jamanetworkopen.2021.25572

**Published:** 2021-09-01

**Authors:** Sten H. Vermund

**Affiliations:** Office of the Dean, Yale School of Public Health, New Haven, Connecticut

Parents and children from low- and middle-income countries (LMIC) may have a myriad of health and social challenges that increase the risk of parental death, chronic conditions, or acute serious illnesses in parents or children, or family economic hardships that can result in child abandonment or orphanhood. Such conditions can include HIV/AIDS, COVID-19, physical or mental trauma, undermanaged chronic diseases, mental health disabilities,^1^ civil strife, extreme poverty with food and housing insecurities, and many others. The intergenerational consequence of such parental death or family disruption is that vulnerable children can be orphaned, abandoned, or separated because of war, civil strife, or related to immigration policy, such as at the US-Mexico border. Varying estimates from UNICEF and other sources suggest between 140 million to 153 million children are orphaned worldwide; orphaned and separated children and adolescents represent at least 7% of the world’s 2.2 billion children under the age of 18 years.

In the article by Braitstein et al,^2^ a research team from Canada, Kenya, and the US reports results of their 10-year cohort study of 2551 orphaned and separated children and adolescents in Uasin Gishu County in northwest Kenya. The Orphaned and Separated Children’s Assessments Related to their Health and Well-Being (OSCAR) Project enrolled children who lived in a variety of housing settings and living circumstances: 1230 children lived in charitable children’s institutions (CCI), 1230 children lived in family-based settings, and 91 children were deemed street-connected youth. The median follow-up time was 6.2 years (interquartile range, 2.0-7.4 years) and differed by venue: 3.1 years (1.0-7.0 years) for participants in CCI institutional care; 6.9 years (2.3-7.4 years) for participants in family-based care; and 6.5 years (2.0-8.1 years) for street-connected youth. Outcomes included death, incident HIV in children who were not infected perinatally, and survival for those children living with HIV.

Many factors can influence whether orphaned and separated children and adolescents are placed in residential or family-based care or must provide for themselves on the street. Random allocation was not possible, so the OSCAR Project investigators used survival regression models to assess associations of care environment with the outcomes. After adjusting for baseline HIV status, age, and sex, youth who lived in a residential CCI setting had a lower adjusted hazard ratio for mortality (AHR, 0.26; 95% CI, 0.07-1.02) compared with youth living in family-based care, but CIs indicated that differences may have been because of chance. Incident HIV was similar for youth living in CCI and youth living in family-based care (AHR, 1.49; 95% CI, 0.46-4.83) with 59 incident HIV infections among the 2551 participants. The HIV incidence rate was 2.06 per 1000 person-years (95% CI, 1.1-3.0 per 1000 person-years) overall, differing by venue (CCI dwellers: 2.2 per 1000 person-years [95% CI, 0.5-3.8 per 1000 person-years]; orphaned and separated children and adolescents living with families: 1.2 per 1000 person-years [95% CI, 0.2-2.1 per 1000 person-years]; and children living on the streets: 15.5 per 1000 person-years [95% CI, 3.1-27.1 per 1000 person-years]). The findings that street-connected youth were far more likely to die or acquire HIV were expected. A conservative conclusion is that the institutionalized CCI youth in Kenya did no worse than youth in family-based settings; the evidence trend suggested that CCI youth may have done better in terms of mortality risk.

Given resource constraints and the number of children requiring care in LMICs, orphaned and separated children and adolescents typically have worse health outcomes than children growing up with biological or adoptive parents.^3,4^ However, it has been unclear whether institutionalized orphaned and separated children and adolescents are better or worse off than those placed in family settings. The Positive Outcomes for Orphans (POFO) Study followed 2013 children (923 institution- and 1090 community-based) for 3 years from 5 LMICs (ie, Kenya, Tanzania, Ethiopia, Cambodia, and India). Potentially traumatic events were equally likely in institutionalized vs home-based settings and quality of care was the dominant factor associated with the outcome, not venue of abode.^5,6^ Findings of the decade-long Kenyan OSCAR Project is compatible with the 3-year, 5-nation POFO Study in that CCI vs family-based venue was not a notable associated with outcomes.

The institutionalized CCI setting of Kenya may have had advantages in improved orphaned and separated children and adolescents health outcomes compared with family-based settings in the Kenyan OSCAR Project.^2,7^ It has been posited that favorable mental health outcomes may be more efficiently nurtured in institutionalized settings vs disparate family-based settings.^7^ Similarly, institutionalized care settings in the 5-nation POFO Study had similar health outcomes to those observed in family-based settings.^5,6^ However, generalizability is elusive, and findings may not extend to orphaned and separated children and adolescents in disparate social settings and well-known institutional abuse of children in past decades. Such settings have seen the consequences of the reuse of needles/syringes, neglect of children’s health and nutrition, and failure to intervene in physical, sexual, or emotional abuse.^8-11^ It is vital to know whether institutionalized living circumstances (eg, CCI in Kenya) are an asset or a debit for the health of these children in specific local conditions. If institutions are humane, enlightened, and adequate funded, they could be equal or superior options for care for orphaned and separated children and adolescents than family-based services. Overwhelming evidence of the toxic nature of life on the street illustrates the importance of research on the impact of quality institutional or family-based services on health outcomes for this population. Evidence-based guidance from social work and public policy research is needed within local contexts as to how to prevent dissolution of families, avoid children living on the streets, and engage homeless children. Braitstein et al^2^ confirmed the very severe disadvantages for children living in the street, and further demonstrated that the children who are in CCI care did not do worse for major health outcomes than children in family-based settings–and may have done slightly better–vital national information for Kenyan child policy.

Global efforts to reduce destitution by enhancing economic and educational opportunities and improving health through preventive and curative services can reduce the number of orphaned and separated children and adolescents. Reducing parental death within families, helping families care for children with special needs, and assisting children with access to educational and health resources can help mitigate harms in all venues, family-based or institutional. Children living on the street are the most vulnerable, by far, so it is nearly certain that reasonable quality institutional or family-based services are far superior options to the brutal conditions of child isolation and homelessness for orphaned and separated children and adolescents.

## References

[1] Wainberg ML, Helpman L, Duarte CS, Curtailing the communicability of psychiatric disorders. Lancet Psychiatry. 2018;5(11):940–944. doi:10.1016/S2215-0366(18)30342-0 30316807 PMC6433373

[2] Braitstein P, DeLong A, Ayuku D, Association of care environment with HIV incidence and death among orphaned, separated, and street-connected children and adolescents in western Kenya. JAMA Netw Open. 2021;4(9):e2125365. doi:10.1001/jamanetworkopen.2021.25365 34529063 PMC8446813

[3] Finlay JE, Fink G, McCoy DC, Stunting risk of orphans by caregiver and living arrangement in low-income and middle-income countries. J Epidemiol Community Health. 2016;70(8):784–790. doi:10.1136/jech-2015-206346 26826211

[4] Raymond JM, Zolnikov TR. AIDS-affected orphans in sub-Saharan Africa: a scoping review on outcome differences in rural and urban environments. AIDS Behav. 2018;22(10):3429–3441. doi:10.1007/s10461-018-2134-1 29721717

[5] Gray CL, Pence BW, Ostermann J, Prevalence and incidence of traumatic experiences among orphans in institutional and family-based settings in 5 low- and middle-income countries: a longitudinal study. Glob Health Sci Pract. 2015;3(3):395–404. doi:10.9745/GHSP-D-15-00093 26374801 PMC4570014

[6] Huynh HV, Limber SP, Gray CL, Factors affecting the psychosocial well-being of orphan and separated children in five low- and middle-income countries: Which is more important, quality of care or care setting? PLoS One. 2019;14(6):e0218100. doi:10.1371/journal.pone.0218100 31194781 PMC6563974

[7] Wilson-Barthes M, Chrysanthopoulou SA, Atwoli L, Ayuku D, Braitstein P, Galárraga O. Cost-effectiveness of care environments for improving the mental health of orphaned and separated children and adolescents in Kenya. J Ment Health Policy Econ. 2021;24(2):31–41. 34151779

[8] Gershoff ET. School corporal punishment in global perspective: prevalence, outcomes, and efforts at intervention. Psychol Health Med. 2017;22(sup1):224–239. 28064515 10.1080/13548506.2016.1271955 PMC5560991

[9] Running Bear U, Thayer ZM, Croy CD, Kaufman CE, Manson SM; AI-SUPERPFP Team. The impact of individual and parental American Indian boarding school attendance on chronic physical health of Northern Plains tribes. Fam Community Health. 2019;42(1):1–7. doi:10.1097/FCH.0000000000000205 30431464 PMC6241300

[10] Zephier Olson MD, Dombrowski K. A systematic review of indian boarding schools and attachment in the context of substance use studies of Native Americans. J Racial Ethn Health Disparities. 2020;7(1):62–71. doi:10.1007/s40615-019-00634-4 31452147

[11] Hersh BS, Popovici F, Jezek Z, Risk factors for HIV infection among abandoned Romanian children. AIDS. 1993;7(12):1617–1624. doi:10.1097/00002030-199312000-00012 8286071

